# Psychopathology predicts the outcome of medial branch blocks with corticosteroid for chronic axial low back or cervical pain: a prospective cohort study

**DOI:** 10.1186/1471-2474-10-22

**Published:** 2009-02-16

**Authors:** Ajay D Wasan, Robert N Jamison, Loc Pham, Naveen Tipirneni, Srdjan S Nedeljkovic, Jeffrey N Katz

**Affiliations:** 1 Department of Anesthesiology, Perioperative and Pain Medicine, Brigham & Women's Hospital and Harvard Medical School, Boston, MA, USA; 2 Department of Psychiatry, Brigham and Women's Hospital and Harvard Medical School, Boston, USA; 3 Millennium Pain Center, Bloomington, IN, USA; 4 Department of Orthopaedic Surgery and Division of Rheumatology, Immunology and Allergy, Brigham and Women's Hospital and Harvard Medical School, Boston, MA, USA

## Abstract

**Background:**

Comorbid psychopathology is an important predictor of poor outcome for many types of treatments for back or neck pain. But it is unknown if this applies to the results of medial branch blocks (MBBs) for chronic low back or neck pain, which involves injecting the medial branch of the dorsal ramus nerves that innervate the facet joints. The objective of this study was to determine whether high levels of psychopathology are predictive of pain relief after MBB injections in the lumbar or cervical spine.

**Methods:**

This was a prospective cohort study. Consecutive patients in a pain medicine practice undergoing MBBs of the lumbar or cervical facets with corticosteroids were recruited to participate. Subjects were selected for a MBB based on operationalized selection criteria and the procedure was performed in a standardized manner. Subjects completed the Brief Pain Inventory (BPI) and the Hospital Anxiety and Depression Scale (HADS) just prior to the procedure and at one-month follow up. Scores on the HADS classified the subjects into three groups based on psychiatric symptoms, which formed the primary predictor variable: *Low*, *Moderate*, or *High *levels of psychopathology. The primary outcome measure was the percent improvement in average daily pain rating one-month following an injection. Analysis of variance and chi-square were used to analyze the analgesia and functional rating differences between groups, and to perform a responder analysis.

**Results:**

Eighty six (86) subjects completed the study. The *Low *psychopathology group (n = 37) reported a mean of 23% improvement in pain at one-month while the *High *psychopathology group (n = 29) reported a mean worsening of -5.8% in pain (p < .001). Forty five percent (45%) of the *Low *group had at least 30% improvement in pain versus 10% in the *High *group (p < .001). Using an analysis of covariance, no baseline demographic, social, or medical variables were significant predictors of pain improvement, nor did they mitigate the effect of psychopathology on the outcome.

**Conclusion:**

Psychiatric comorbidity is associated with diminished pain relief after a MBB injection performed with steroid at one-month follow-up. These findings illustrate the importance of assessing comorbid psychopathology as part of a spine care evaluation.

## Background

Facet injections are among the most commonly performed non-surgical procedures in the United States for axial low back or neck pain, representing a range of techniques and indications[1] Published reviews of insurance claims data from the U.S. have been unable to determine the frequency by which each injection technique is used (intrarticular vs. MBB), whether they were performed for diagnostic or therapeutic purposes, or what medications were injected[1,2] It is likely that the entire spectrum of indications, techniques, and medications are used with significant frequency, although some have recommended that certain approaches are preferable[3] While there is heterogeneity in the manner in which they are being used, identification of predictive factors in order to help determine success from these injections as a treatment for back pain is needed.

There is little agreement on what factors predict a successful outcome from facet injections and much depends on how they are used: indication (diagnostic vs. therapeutic), method (intrarticular vs. MBB), or medications (anesthetic only vs. steroid)[4] Low-volume intrarticular injections are more specific for diagnosing facet-mediated pain, while MBBs are more useful in treating a wider range of back or neck pain since they block other potential pain generators such as the multifidus muscle and the interspinales ligament[5,6] The diagnostic and therapeutic results have been comparable between the two techniques (intrarticular vs. MBB),[7,8] and both are associated with significant rates of false positives and negatives[4] Some clinicians have used the results of these blocks in deciding whom to offer a radiofrequency lesioning (RFL) procedure, which may confer longer benefit. This has been reported in some controlled studies as an average of 50% pain reduction lasting 6 months[9,10] Since an RFL of the medial branch dorsal ramus nerve also denervates the medial third of the multifidus muscle[11], there is a rationale supporting the MBB method, either for therapeutic or prognostic reasons.

An initial controlled trial found little therapeutic efficacy at six weeks for facet injections with corticosteroid[12] But more recent systematic reviews conclude that there is moderate evidence for significant benefit with either the intrarticular or MBB facet block technique (at least 30% pain relief at 3 months, Level III evidence)[13] In many of these reviewed studies injectate volumes of 1–2 mls were used, which then are likely to spread beyond the dorsal ramus nerve to adjacent structures. Thus, even though therapeutic MBBs with steroid are treatments that are not specific to the facet joint, they may be useful in helping patients with axial neck or back pain.

There is, however, great variability in the subjects' responses to MBBs[13] Few predictive factors for success have been noted consistently, except for positive SPECT bone scan findings of facet disease[14] While earlier studies suggest that response to facet injections could be predicted by patient history and physical examination,[15,16] subsequent work has shown that neither history, physical exam, nor radiographic findings (CT, MRI, or X-ray) can predict pain relief[4,17-21]

Because symptoms and anatomic findings are poorly predictive of results, it is difficult for clinicians to appropriately select patients for MBB injections. Although MBBs would not be indicated for everyone with axial pain, overly stringent selection criteria for performing these injections would likely exclude many who might otherwise benefit from this treatment. Yet, subgroups of responders in pain treatment studies of clinic populations can be identified reliably utilizing prospective observational designs based on possible predictive factors and applying operational inclusion criteria[22] This suggests that in treatment studies of MBBs confounding factors such as the lack of a clear physical diagnosis and the lack of specificity of an MBB to block the facet joint can be mitigated. Further identification of appropriate selection criteria for a MBB would allow for improved predictability of outcome.

Psychiatric illness–most often marked by depression, anxiety, and personality pathology–has been shown to be a significant predictor of treatment outcome for chronic musculoskeletal pain,[23] regardless of whether it occurs prior to or after developing chronic pain[24] Psychopathology afflicts 50–75% of clinic populations of chronic pain patients,[25,26] and those with a combination of negative affective disorders (such as depression and anxiety, which often occur together) are prone to the worst outcomes[27] In an effort to examine the relationship between psychiatric comorbidity and the diagnostic use of facet injections, Manchikanti, et. al., [28] identified 100 chronic low back pain patients with and without somatization, who underwent intrarticular facet injections with local anesthetic only, using the double block method. They reported that the rate of immediate pain relief and positive response from two blocks was the same (42%) whether a somatization disorder was present or not. However, the non-somatizers also had significant rates of major depression and generalized anxiety disorder, and 78% of the entire study population had at least one major psychiatric disorder. Also, the patients were only assessed for immediate pain relief after the procedure and were not followed. Furthermore, conscious sedation was used for the procedures, which the authors have reported to be a significant confounder of positive and negative responses[29,30] Thus, while the Manchikanti et al., study reported little relationship between psychopathology and outcome from diagnostic facet injections, there were confounding factors in the study design that made the results difficult to interpret and therapeutic benefit was not studied.

In sum, psychiatric comorbidity is a negative predictor of treatment outcome in general for chronic musculoskeletal pain and the relationship between facet injection results and psychiatric illness is still unclear. The aim of this study was to evaluate whether psychiatric comorbidity predicted the outcomes of MBBs for patients with axial back or neck pain. We hypothesized that patients with high levels of depression and anxiety symptoms would have a diminished response to a therapeutic MBB compared with those with little psychiatric comorbidity.

## Methods

### Design and setting

This was a prospective, observational cohort study done in a single, large, urban, university-based pain management center. After IRB approval from Brigham and Women's Hospital, all patients undergoing lumbar or cervical MBB injections were invited to participate. Data collection consisted of questionnaire surveys and de-identified medical records review. Seven physicians, all of whom were board-certified in Pain Medicine and had practiced together for at least 5 years, participated in this study.

### Inclusion criteria

After giving verbal consent, subjects were considered for inclusion based on a decision by the treating physician to perform an MBB either as an independent therapy or as a prerequisite for a possible RFL. Patients were selected for an MBB based on the physician's assessment, which included: 1) obtaining a history of primarily chronic axial neck or low back pain, with radiation of pain in a common pattern for facet syndrome,[31,32] 2) documentation of whether the patient had facet loading signs or paraspinal tenderness,[33] and 3) a review of relevant supporting radiographic studies suggesting facet arthropathy (with the understanding that patients frequently have mixed conditions such as concurrent disc disease or spinal stenosis)[4] These operational criteria have been criticized in making the difficult diagnosis of facet-mediated pain[4] It was not our intention to use these common criteria to diagnose whether our patients had facet syndrome. However, by applying certain selection criteria uniformly, our study would be able to evaluate the results of the intended procedure for a certain group of patients with axial back pain.

### Procedures

At the time of the initial evaluation a comprehensive treatment plan was proposed, which may have included the use of injections as well as multidisciplinary treatments of medications, physical therapy, and behavioral medicine, if indicated. The MBB procedure was performed at three or four levels in the cervical or lumbar spine, using local anesthetic at each level (1–2 mls of 0.5% lidocaine for skin and subcutaneous infiltration) and without any sedation. A 22 gauge spinal needle was then placed at each spinal level using flourscopic guidance, so that each needle was positioned at the junction of the transverse process and the superior articular process for lumbar injections and at the waist of the articular pillar for cervical injections. The injections consisted of methylprednisolone (20–30 mg, .5–.75 mls) and 0.25% bupivacaine (.5 ml) at each level, a total of 1.0–1.25 mls injectate volume per level. Depending on the physician's assessment of the patient's condition, injections were done unilaterally or bilaterally at 3–4 spinal levels. In all cases a total of 120 mg of methylprednisolone was injected.

### Data collection

All measures were administered before the procedure and one-month afterwards. For patients who did not schedule a one-month follow-up visit, the scales were mailed after three weeks for them to complete and return. For those who missed their one-month visit, the scales were mailed to them that day.

### Primary outcome

Percent improvement in average daily pain rating at one month was calculated using item 5 (average daily pain rating, 0–10 scale) of the Brief Pain Inventory (BPI)[34] The BPI is a 15 item questionnaire assessing pain location, intensity, relief, and quality, as well as pain-related quality of life, validated in cancer and non-cancer pain studies[35] The activity interference items, such as pain interference with activity, sleep, or work, have shown a high correlation with other functional and quality of life measures, such as the SF-36[36]

### Primary predictor of outcome

The overall level of combined depression and anxiety symptoms (*High*, *Moderate*, or *Low *levels of psychopathology) was the primary predictor. The treating physicians were blinded to group assignment. Group assignment was determined using the Hospital Anxiety and Depression Scale (HADS),[37] which includes subscale scores for anxiety and depression. The HADS is a 14-item self-report survey designed for populations with medical illness. It does not include somatic symptoms, such as fatigue and sleeplessness, which may otherwise be attributable to physical illness. It has been validated in several medical illness populations with a sensitivity and specificity of .66–.97 for a DSM-IV major depression or generalized anxiety disorder diagnosis[38] Using validated cutoff points, those with anxiety or depression subscale symptom scores of ≥ 9 were classified as having high anxiety or depression respectively, which most likely reflects a comorbid anxiety or depression disorder[38] Those with anxiety or depression subscale scores ≤ 6 were classified as having low anxiety or depression levels, i.e. those significantly less likely to have a disorder. Moderate levels for anxiety or depression were operationalized as including all of the scores in between these cutoff points. Patients were assigned to *High*, *Moderate*, or *Low *level of psychiatric comorbidity based on the subscale scores of the HADS. To be in the *High *group, scores had to be high on both the depression and anxiety subscales (i.e., at least 9 on each, HADS total score ≥ 18). To be in the *Low *group, scores had to be low on both subscales (total score ≤ 12), and the *Moderate *group was all others not meeting *High *or *Low *criteria. This approach emphasizes the magnitude of difference in total negative affective symptoms between the *Low *and *High *groups and has been used in previous studies by the authors[23,39]

### Sample size calculations

We used a sample size calculation for a comparison of means of the primary outcome between the three groups. With Type I error of 5%, we sought 80% power to detect a 30% difference in pain improvement between the *Low *and *High *groups. This is considered a clinically meaningful difference[40] Using data from a previous study performed by the authors in a similar clinic population,[23] we assumed an average baseline pain level of 6.1 and a standard deviation of 1.4. We estimated that 80 subjects would be required and recruitment stopped when approximately 80 one-month follow-up surveys had been returned.

### Data Analysis

An analysis of variance (ANOVA) was used to characterize the relationship between the primary predictor and outcome. Chi-square analysis was used to perform a responder's analysis of patients categorized as those with 1) at least 30% and 2) at least 20% improvement in pain. Twenty percent improvement was evaluated to examine whether the response trends were consistent between the groups at various levels of improvement. Baseline demographic, social, and pain history variables were included in an analysis of covariance (ANCOVA). The data used to calculate sample size requirements[23] led us to anticipate that the *High *group would be less likely to be working or married, and more likely to be taking prescription opioids. We also included as covariates which physician performed the procedure and whether other treatments were begun at the time of the initial evaluation (such as non-opioid medications or physical therapy).

## Results

One hundred sixty six (N = 166) subjects were recruited and follow-up surveys were returned by 86 of 166 participants (52% response rate). No differences were found between the survey responders and nonresponders on any demographic, social, pain history, or psychiatric variables.

The demographic, social, psychiatric, and pain history characteristics of those who had completed the 1-month follow-up surveys are presented in Table 1. 29 of the subjects (34%) were grouped as *High *psychopathology, 20 of the subjects (23%) were in the *Moderate *psychopathology group, and 37 (43%) were in the *Low *psychopathology group. There was a significant correlation between levels of depression and anxiety (Pearson correlation coefficient = .64, p = .01). Of the survey responders, the *High *psychopathology group (n = 29) had a significantly lower percentage working (53%) and a significantly higher percentage (73%) taking prescription opioids (p < .05).

**Table 1:** Demographics, Social, Psychiatric, and Pain History

	***Psychopathology Group *(N = 86)**			
***Variable***	**Low (n = 37)**	**Mod (n = 20)**	**High (n = 29)**	**Sig**^1^
**Age **(mean, yrs.)(CI)^4^	64.0 (59.0–69.0)	56.4 (49.2–63.6)	58.7 (53.1–64.2)	*NS*
**Sex **(% female)	59	76	63	*NS*
**Work Status **(% working)^2^	87	95	53	**p = .003**
**WC/Lit **(%yes)^3^	5	5	3	*NS*
**Maritial Status **(% married)	69	44	59	*NS*
**Pain Duration **(mean, yrs)(CI)^4^	6.1 (3.7–8.6)	7.9 (4.4–11.4)	7.1 (4.4–9.8)	NS
**Location **(% low back)	82	90	93	*NS*
**Previous Spine Surgery **(% yes)	39	24	40	*NS*
**Hx Smoking **(% yes)	54	62	47	*NS*
**Prescription Opioids **(% yes)	36	33	73	**p = .003**
**Baseline Pain Level **(0–10)(CI)^4^	6.1 (5.6–6.7)	6.8 (6.0–7.5)	6.2 (5.6–6.8)	*NS*
**Facet Loading Signs **(% yes)	38	35	22	*NS*
**Paraspinal Tenderness **(%yes)	44	47	48	*NS*
**Depression Score **(mean)(CI)^4^	4.6 (3.9–5.3)	7.7 (6.7–8.6)	11.6 (10.8–12.4)	**p = .0001**
**Anxiety Score **(mean)(CI)^4^	4.8 (3.9–5.8)	8.3 (7.0–9.6)	13.2 (12.1–14.3)	**p = .0001**

1 = Chi square and ANOVA analysis

2 = Working includes homemakers taking care of children and those retired

3 = Active workers' compensation or injury litigation case (unsettled)

4 = 95% Confidence Interval

NS = nonsignificant

**Figure 1:**
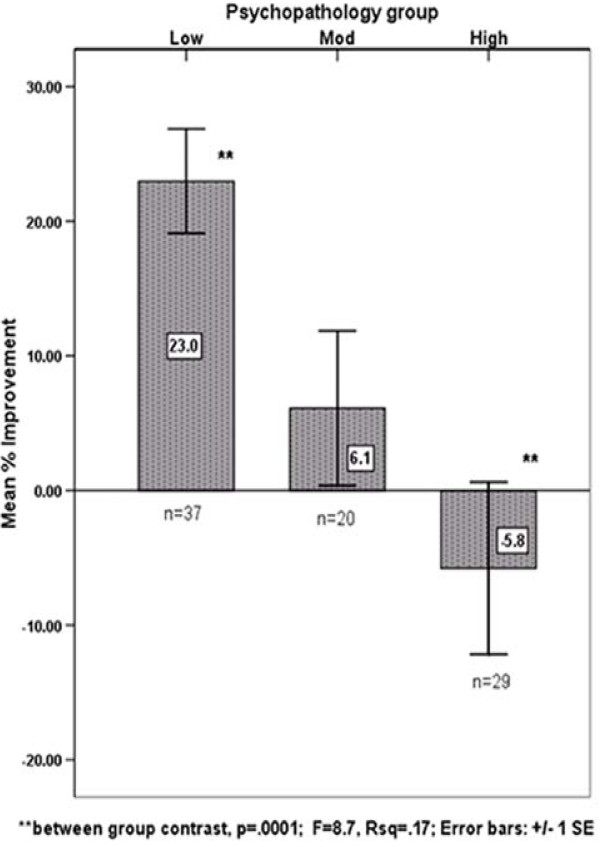
**Psychopathology Group and Pain Improvement**. **between group contrast, p = .0001; F = 8.7, Rsq = .17; Error bars: +/- 1 SE

Figure 1 summarizes the results for the main outcome variable, the percent improvement in average daily pain rating at one-month follow-up for each psychopathology group. There were significant differences amongst the groups (ANOVA, R^2 ^= .17, p < .001), with the *Low *(23% improvement) vs. *High *(-5.8% worsening) groups having the most significant contrast between group comparison (Tukey correction; p < 0.0001). None of the three groups reported significant improvements in the pain interference items of the BPI (activity change, sleep change, etc.) at one month follow-up. Nor were there significant changes in anxiety or depression ratings after one month.

**Figure 2:**
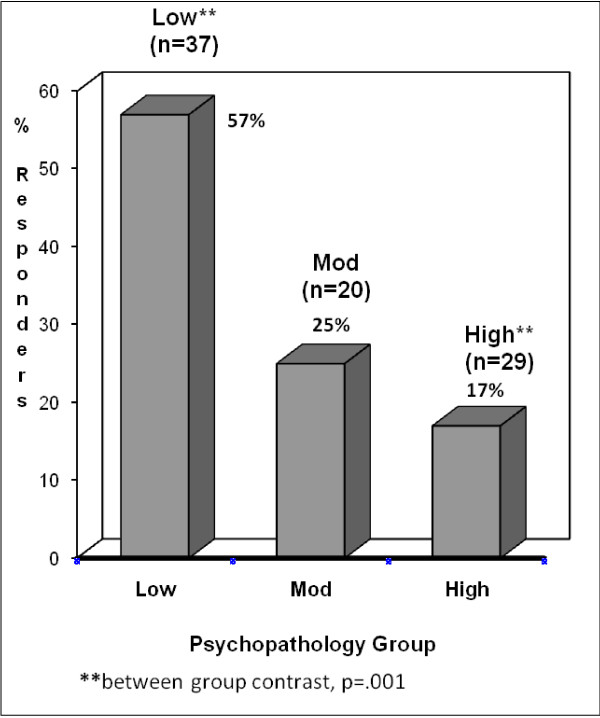
**Percentage of Patients in Each Group with 20% Improvement**. **between group contrast, p = .001

**Figure 3:**
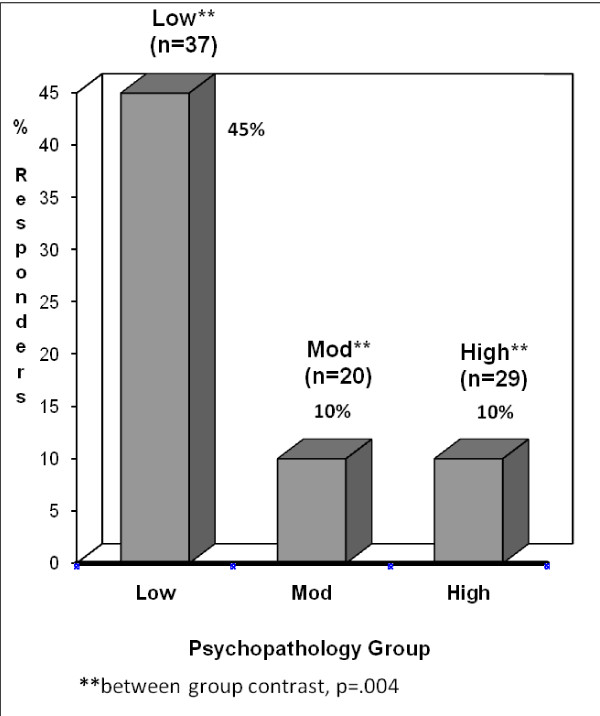
**Percentage of Patients in Each Group with 30% Improvement**. **between group contrast, p = .004

Figures 2 and 3 display a responder analysis of the proportion of each group with at least 20% or 30% improvement in pain levels, and demonstrates more contrasts between groups. For those with at least 30% improvement, the *Low *psychiatric morbidity group had a significantly greater percentage of patients with pain relief than either the *Moderate *or *High *groups. When the criterion was set at 20% improvement, the same trends were found.

ANCOVA was used to test whether specific covariates altered the strength of the predictive relationship between psychopathology level and the degree of pain improvement from a MBB. First, separate univariate tests with percent pain improvement as the outcome were run for each of the baseline factors listed in Table 1, treating physician, and concurrent treatments (e.g. medications). Only opioid use was a significant predictor (associated with less pain improvement, Beta = -14; p < 0.05) and was carried over to the next stage of modeling. Psychopathology group and opioid use were then both entered into the model to test whether opioid use remained a significant main effect, whether it mitigated the strength of psychopathology group as a primary predictor, and whether there was a significant interaction between the two. Opioid use was not found to be a significant predictor, nor were the estimated marginal means for psychopathology group altered by > 10% compared with the means in the model with psychopathology group as the only predictor. Psychopathology group remained as the only significant predictor of outcome (*F *= 6.0, R^2 ^= .18, p < 0.01), and no significant interaction was found.

## Discussion

This study examined the impact of psychiatric comorbidity on the response to medial branch blocks for spinal pain and demonstrated that patients with elevated levels of depression and anxiety are less likely to have a positive outcome after a MBB injection performed for axial low back or neck pain. One month following a MBB, the *High *psychopathology group reported a 5% increase in pain from baseline, while the *Low *psychopathology group on average reported a 23% improvement in pain intensity. These differences are even more apparent in the responder analysis, in which the *Low *group had 2 to 4 times the rate of positive response (45–57%) than the *High *and *Moderate *groups (10–25%). Neither group showed significant improvements in function. The degree of improvement in the *Low *group was less than what has been reported in previous studies[13] We think this occurred primarily for two reasons: 1) Many of the earlier studies were performed in community-based pain clinics whose patients tend to have less severe pain and a shorter duration of pain, and thus are less likely to be refractory to treatment than our patient sample from a tertiary care center. 2) Some of the studies of facet injections using steroid did so after a positive response to a facet injection with anesthetic only. Hence due to selection bias in some of the previous studies, the study populations are not directly comparable to our subjects.

The *Low *and *High *psychopathology groups had clear contrasts in psychiatric symptoms, with levels in the *High *group consistent with major depression or generalized anxiety disorder diagnoses. Forty eight percent of the sample had either a high level of depression or anxiety. This is consistent with previous samples from pain clinics reporting a prevalence of psychiatric comorbidity ranging from 40–80%[41]

There is mounting evidence from neuroimaging studies that psychiatric overlay in chronic painful illnesses alters pain processing in the "pain matrix" in the brain[42,43] This may result in amplified pain perception (particularly, the affective component), akin to neuropathic mechanisms amplifying pain at the level of the spinal cord. These studies support the view that comorbid psychiatric illness may disrupt descending pan inhibition, whose system originates in brain areas modulating pain and mood, such as the insula and anterior cingulate gyrus[44] This alteration in endogenous antinociceptive activity may undermine the effectiveness of MBBs modulating pain transmission at the spinal level. Alternatively, one should consider that adverse motivational/behavioral factors in the *High *group undermined any analgesic benefit of the MBB steroid injection.

These results suggest, however, that use of careful selection criteria may increase the likelihood that patients may benefit from steroid MBB injections for axial back or neck pain. Psychiatric assessment is an important component of any comprehensive spine care evaluation or treatment[45] The administration of the HADS was feasible in our medical setting and practitioners may consider using it as part of the initial evaluation, upon which a treatment plan is constructed. We are not suggesting that high depression or anxiety scores be used as exclusion criteria for an MBB, if that is considered. Rather, high scores may signal that a multidisciplinary treatment plan should begin first and then an MBB may be considered if that patient adapts better psychologically to their pain. This hypothesis remains to be tested.

Furthermore, psychiatric comorbidity is easily identifiable and inherently treatable in a medical setting, and the majority of psychiatric care in the United States is successfully delivered by primary care physicians utilizing medications and/or psychotherapy[46] Even subthreshold levels of depression or anxiety respond to medication treatment,[47,48] suggesting that patients with moderate psychopathology in this study can also be readily treated by the spine care physician.

While high levels of depression and anxiety were found to predict response to steroid MBB injections, just as interesting is that no other psychosocial (e.g., work status) or medical variables (e.g., opioid use) were found to have a significant impact on outcome. Recently, Cohen and colleagues reported that paraspinal tenderness predicted a positive response to RFL[49] The results of our study could not confirm that physical exam findings predicted response to a single-injection MBB procedure. Although our results comport with the findings of other studies associating psychological variables to results from chronic spinal pain treatments,[50,51] replication of these results is needed.

This study included a heterogeneous group of back pain patients, such as those with concurrent disc disease or spinal stenosis, and a MBB has been used by some as an aid to diagnose facet syndrome. Even though this study was not designed to confirm a diagnosis, the selection criteria for inclusion in the study made it less likely that these patients had radicular pain due to nerve root involvement or nonspecific low back or neck pain[4] Given the lack of differences in physical exam findings between groups, it is also unlikely that differences in spine pathology accounted for the study findings amongst the psychopathology groups. In sum, most probably the heterogeneity in spine pathology was distributed equally amongst the groups, and thus is less likely to be a significant confounder to our results.

Another goal of a MBB, other than to clarifying a diagnosis, is to identify suitable patients for RFL. Commonly this is done by judging the short-term response to a MBB, using techniques such as the double-block method to reduce the incidence of false-positive responses. Since we were most interested in the one-month outcome after a therapeutic MBB with steroid in a group of uniformly selected patients, we did not utilize the double-block technique to determine candidacy for RFL, although this procedure might be considered in future studies. The design of this study did not offer the opportunity to evaluate the potential outcome from RFL after a steroid MBB, although future studies would be useful in determining whether treatment of psychiatric comorbidity prior to or in conjunction with doing an MBB would improve the response rate to an RFL. Furthermore, we cannot determine whether the positive results in the *Low *group were placebo responses. But because the patients were treated with an MBB without the physicians knowing which group they were in, the nonspecific effects of treatment were minimized and the incidence of false-positive responses to the MBB procedure would likely be the same in our study groups.

Several additional limitations of this study merit discussion: First, we were able to follow-up only 52% of the participants, and consequently, although not statistically significant, selection bias may have influenced the results. Next, unlike some previous studies, we did not focus on whether response to a MBB was useful in diagnosing facet-mediated pain. Rather, in a subtle yet salient departure, we concentrated on whether psychiatric factors predicted response to a therapeutic MBB with corticosteroid performed in patients with axial neck or low back pain. This is an equally relevant clinical issue, and as stated, this is a common indication for a MBB. We may have obtained different results had we designed the study as a diagnostic-based study rather than a treatment-based study. And lastly, we only collected 1-month follow-up data. It is possible that more frequent ratings over a longer period of time could have given different results.

## Conclusion

Despite these shortcomings, the results of this study suggest that high levels of psychopathology have a negative impact on MBB outcome. Importantly, no other medical or psychosocial covariates affected this result. Future studies should examine whether these findings extend to those undergoing RFL. It will also be fruitful to examine whether appropriate treatment of anxiety and depression in a group of patients with chronic pain improves the results of MBB injections and other procedural therapies.

## Competing interests

The authors declare that they have no competing interests.

## Authors' contributions

ADW was responsible for study design, data collection and analysis, and manuscript preparation. RNJ was responsible for study design and analysis and manuscript editing. LP was responsible for data collection. NP was responsible for data collection. SSN was responsible for data collection and manuscript editing. JNK was responsible for study design and analysis and manuscript editing. All authors read and approved the final manuscript.

## Pre-publication history

The pre-publication history for this paper can be accessed here:


